# The Endophytic Bacterial Microbiota Associated with Sweet Sorghum (*Sorghum bicolor*) Is Modulated by the Application of Chemical N Fertilizer to the Field

**DOI:** 10.1155/2018/7403670

**Published:** 2018-09-30

**Authors:** Cintia Mareque, Thais Freitas da Silva, Renata Estebanez Vollú, Martín Beracochea, Lucy Seldin, Federico Battistoni

**Affiliations:** ^1^Departamento de Bioquímica y Genómica Microbianas, Instituto de Investigaciones Biológica Clemente Estable, Avenida Italia 3318, Montevideo 11600, Uruguay; ^2^Laboratório de Genética Microbiana, Instituto de Microbiologia Paulo de Góes, Universidade Federal do Rio de Janeiro (UFRJ), Rio de Janeiro, Brazil

## Abstract

Sweet sorghum (*Sorghum bicolor*) is a multipurpose crop used as a feedstock to produce bioethanol, sugar, energy, and animal feed. However, it requires high levels of N fertilizer application to achieve the optimal growth, which causes environmental degradation. Bacterial endophytes, which live inside plant tissues, play a key role in the health and productivity of their host. This particular community may be influenced by different agronomical practices. The aim of the work was to evaluate the effects of N fertilization on the structure, diversity, abundance, and composition of endophytic and diazotrophic bacterial community associated with field-grown sweet sorghum. PCR-DGGE, quantitative PCR, and high-throughput sequencing were performed based on the amplification of *rrs* and *nifH* genes. The level of N fertilization affected the structure and abundance but not the diversity of the endophytic bacterial communities associated with sweet sorghum plants. This effect was pronounced in the roots of both bacterial communities analyzed and may depend on the physiological state of the plants. Specific bacterial classes and genera increased or decreased when the fertilizer was applied. The data obtained here contribute to a better understanding on the effects of agronomical practices on the microbiota associated with this important crop, with the aim to improve its sustainability.

## 1. Introduction

Globally, sweet sorghum (*Sorghum bicolor*) is the fourth most important cereal and is a multipurpose crop that is used in grain, forage, syrup, fodder, and bioethanol production [1]. Microbial communities play a crucial role in ecosystems, and particularly plant microbiomes can modulate the growth, health, productivity, C-sequestration, and phytoremediation of plants and play a key role in global biogeochemical cycles [2].

Bacterial endophytes are defined as bacteria that can be detected at a particular moment within the internal tissues of an apparently healthy host plant [3]. Many endophytes are likely to have positive effects on their hosts, with the best examples being N_2_-fixing bacteria [4], but the potential of applying endophytic bacteria as inoculants is underexplored [5–7]. Several members of the phyla Proteobacteria, Firmicutes, Bacteriodetes, and Actinobacteria have been isolated as endophytes. These bacteria have been recognized to have profoundly favourable impacts on plant growth by producing phytohormones, synthesizing fungicidal and/or bactericidal substances, enhancing the availability of minerals, possessing phosphate-solubilizing activity, and providing nitrogen to plants [4]. In addition, endophytic bacteria are an effective agent for stimulating plant secondary metabolism and for improving or producing functional components [4, 8]. These features mentioned stress the potential of the endophytic bacteria to be used as a bioinoculant of agronomical important crops, with the aim to develop more sustainable production systems [9].

The structure and diversity of the endophytic communities have been shown to be potentially influenced by several factors, such as the plant species and genotype, agricultural practices, and environmental conditions [10–12]. To better understand and manipulate the contribution of endophytic bacteria to plants when used as bioinoculants, it is crucial to decipher the community structure, diversity, composition, metabolic processes, adaptability, and beneficial features of the microbiome associated with target crops. Plants have been shown to harbour an enormous diversity of bacteria, including diazotrophs [4, 13, 14]. Therefore, a better understanding of the endophytic bacteria microbiota of host plants may help elucidate their role within their hosts, moving toward the development of more sustainable agronomical practices.

The availability of nitrogen often limits crop productivity, and chemical nitrogen fertilization is widely used to increase the yield of agronomical crops. However, this practice has a high environmental and economic cost, since crop plants are able to use only 50% of the applied fertilizer, while the rest is lost from the plant-soil system through gaseous emissions, runoff erosion, and leaching [15, 16]. The environmental impacts of this loss range from greenhouse effects, ozone layer damage, and acid rain to changes in the global N cycle and nitrate pollution of surface and ground water [17]. In addition, the application of N fertilizer could also inhibit the N_2_-fixation process by diazotrophic bacteria in the soil or associated with the plants [18].

These problems emphasize the urgent need for new technologies based on plant growth-promoting bacteria (PGPB) to help achieve more sustainable agricultural production systems.

Previously, part of the cultivable community associated with sweet sorghum (cv. M81E) was described. Isolates from this community showed several plant growth-promoting (PGP) traits, and some of them were described as plant growth promoters of this sweet sorghum cultivar [19].

The aim of the present study was to evaluate the effects of N fertilization on the structure, diversity, abundance, and composition of endophytic and diazotrophic bacterial communities associated with sweet sorghum in fields. To study these communities, DNA fingerprinting, quantitative PCR, and high-throughput sequencing techniques were used.

## 2. Materials and Methods

### 2.1. Plant Sampling and DNA Extraction

Sweet sorghum plants were sampled from two different fields with contrasting N fertilization levels: 0 and 100 kg N ha^−1^, at the cropping region Bella Union-Artigas, Uruguay (30°37′56.0^″^S 57°21′18.0^″^W). The soil physicochemical characteristics were analyzed as follows: pH 6.2, 42% sand, 25% silt, 33% clay, 1.51% organic matter, and 0.12% total N.

At the laboratory, each collected plant (three plants from each fertilization level) was independently divided into roots and upper and lower stems, which were used for total microbial DNA extraction. The total microbial community DNA was extracted from 1.5 g of each plant fraction using the PowerSoil® DNA Isolation Kit (MO BIO Laboratories, Inc. USA) and purified with the Kit Wizard® DNA Clean-Up System (Promega, USA). Prior to the bacterial DNA extraction, the stem samples were disinfected with 70% EtOH and the epidermis was peeled off with a sterile scalpel. In the case of the roots, the rhizospheric soil was removed by vortexing for 10 min in 0.9% NaCl, the material was disinfected for 10 min in 70% EtOH, surface-sterilized 30 min in 4% sodium hypochlorite, and then rinsed with sterile deionized water. Finally, the root samples were sonicated for 15 min and vortexed for 1 min. Subsequently, the ends of the material were removed with a sterile scalpel and discarded, and sterility tests were conducted on the remainder of the tissue on TSA (trypticase soy agar) plates. DNA preparations were visualized after electrophoresis in a 1% (*w*/*v*) agarose gel in 1x TBE buffer to access their integrity, stained with GoodView (Beijing SBS; Genetech), and stored at −20°C prior to PCR amplification.

### 2.2. Nested PCR Amplification of the *nifH* and 16S rRNA Coding Genes

For DGGE analyses, the 16S rRNA and *nifH* gene sequences from stem and root samples were amplified by triplicate, using nested PCR as previously described [20–22].

For 16S rRNA coding gene amplification, the first PCR was carried out with the forward primer 799F and the reverse primer 1492R (Table S1) [20] generating a product of approximately 700 bp. The second step was carried out with the forward primer F968 containing a GC clamp and the reverse primer R1401 (Table S1) [23] yielding a product of 433 bp. For both reactions, the PCR mixture contained 1.0 *μ*l of template DNA (16 ng *μ*l^−1^), 2x *GoTaq*® Reaction Buffer, 2.1 mM MgCl_2_, 0.4 mM dNTPs, 0.2 *μ*M of each primer, and 2 U *Taq* polymerase, in a final reaction volume of 25 *μ*l. In the second PCR, 1 *μ*l of the first PCR product was used as a template. The PCR conditions were as follows: 35 cycles consisting of denaturing at 94°C for 20 sec, annealing at 53°C for 40 sec for the first PCR and 48°C for 90 sec for the second PCR, and primer extension for 40 sec for the first PCR and 90 sec for the second PCR at 72°C with a final extension at 72°C for 10 min.

DGGE analyses based on the 16S rRNA gene, of the Proteobacteria, Actinobacteria, and Firmicutes phyla, were performed using specific primers for each group (Table S1). The reaction mixtures and the PCR conditions were as previously reported [20, 23–27], while the conditions for the second PCR were the same as mentioned above.

For the *nifH* gene amplification, the first PCR was carried out with the forward primer FGPH19 and the reverse primer POLR (Table S1) [21, 22], generating a product of 429 bp. The second PCR was carried out with the forward primer POLF containing a GC clamp and the reverse primer AQER (Table S1 [21]), yielding a product of 320 bp. The PCR reaction mixture contained 1 *μ*l of template DNA (16 ng *μ*l^−1^), 10 *μ*l 5x *GoTaq*® Reaction Buffer, 2.1 mM MgCl_2_, 0.8 mM dNTPs, 0.2 *μ*M of both sets of primers, and 1.25 U *Taq* polymerase, in a final reaction volume of 50 *μ*l. For the second reaction, 1 *μ*l of the first PCR product was used as a template for the next reaction. The PCR conditions were as follows: 30 cycles consisting of denaturing at 94°C for 1 min, annealing at 50°C for 1 min for the first PCR and 55°C for the second PCR, and primer extension for 2 min at 72°C with a final extension at 72°C for 5 min. The amplification products obtained were analyzed on 1% (*w*/*v*) agarose gels via electrophoresis in TAE buffer (20 mM Tris-acetate, 0.5 mM EDTA; pH 9) and stained with GoodView (Beijing SBS; Genetech).

### 2.3. Denaturing Gradient Gel Electrophoresis (DGGE)

**Figure 1:**
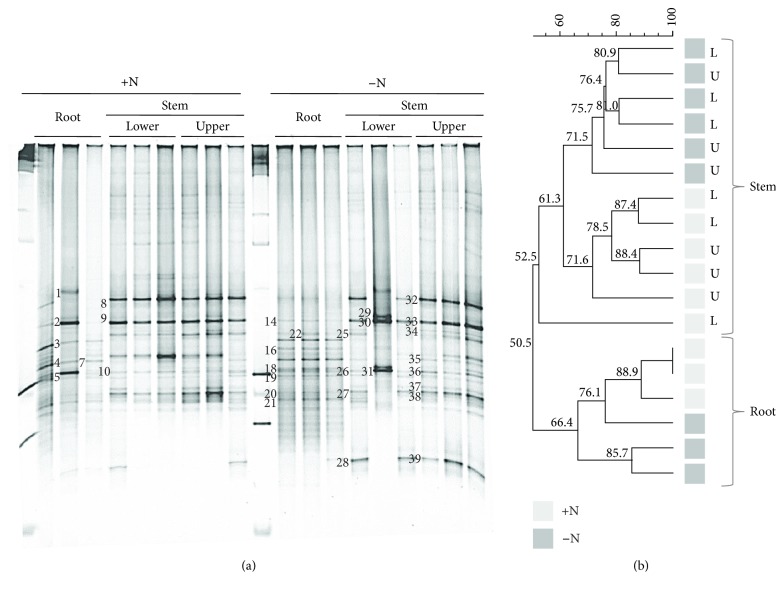
(a) Denaturing gradient gel electrophoresis (DGGE) fingerprints of 16S rRNA gene fragments amplified from endophytic DNA templates isolated from sweet sorghum plants grown under high (+) and low (−) N fertilization levels (100 and 0 kg N ha^−1^, respectively). Numbers indicate the excised bands from which sequences were determined. (b) Dendrogram obtained using the unweighted pair group method with mathematical averages and DICE similarity coefficients. Grey and black squares: high and low nitrogen fertilization levels, respectively. L and U: lower and upper stem parts, respectively.

**Figure 2:**
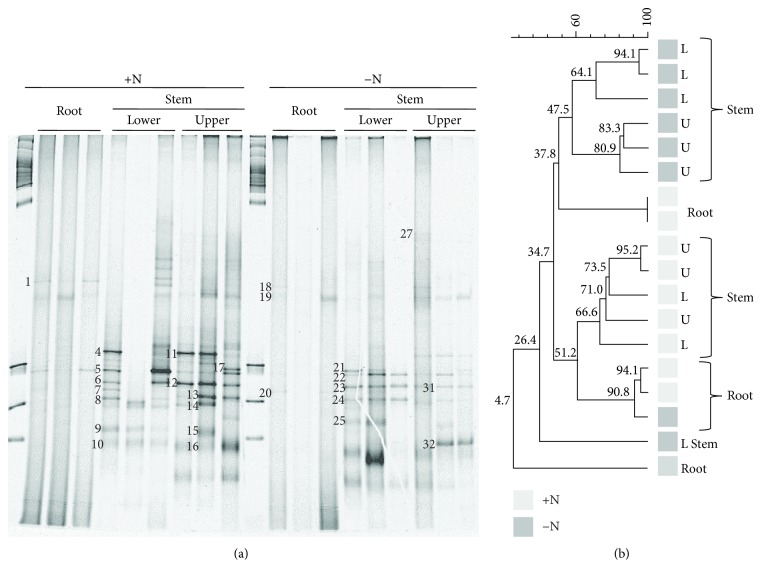
(a) Denaturing gradient gel electrophoresis (DGGE) fingerprints of *nifH* gene fragments amplified from endophytic DNA templates isolated from sweet sorghum plants grown under high (+) and low (−) N fertilization levels (100 and 0 kg N ha^−1^, respectively). Numbers indicate the excised bands from which sequences were determined. (b) Dendrogram obtained using the unweighted pair group method with mathematical averages and DICE similarity coefficients. Grey and black squares: high and low nitrogen fertilization levels, respectively. L and U: lower and upper stem parts, respectively.

PCR product (20 *μ*l) was loaded onto 8% (*w*/*v*) polyacrylamide gels, 1 mm thick, in 1x TAE buffer (20 mM Tris-acetate, 0.5 mM EDTA; pH 9), with a 40–65% urea and formamide denaturant gradient for the study of Alphaproteobacteria, Betaproteobacteria, and Firmicutes, 45–75% for Actinobacteria, and 20–70% for the diazotrophic community. The electrophoresis was run at 60 V for 16 hours using the D-Code system from Bio-Rad Laboratories. The gels were stained for 30 min with 1x SYBR® Green (Invitrogen™) and the bands visualized digitalized using Storm™ (GE Healthcare). Selected bands (numbered in Figures 1 and 2) were excised from the gels with a sterile scalpel, reamplified, and sequenced. For the latter, the bands were eluted at 4°C overnight in 50 *μ*l of Milli-Q water, and 1 *μ*l of each supernatant was used as a PCR template. The reamplification reaction conditions were the same as used for the second cycle described above using the primers F968-GC and R1401 for the 16S rRNA*;* or the primers POLF-GC and AQER for the *nifH* gene. PCR products obtained were sent for sequencing to Macrogen Inc., Korea. Forward and reverse sequences obtained were assembled using the DNA Baser Sequence Assembler v3.x 302 (2010) (http://www.DnaBaser.com). Nucleotide sequences obtained were identified by BLASTn analyses [28], using the GenBank database from the National Center for Biotechnology Information. All sequences obtained in this study were deposited in the GenBank database under the accession numbers: KY062497-KY062552.

### 2.4. Bioinformatics and Statistical Analysis

The dendrograms and the binary matrix based on the digitalized image of the DGGE gels were constructed with the UPGMA algorithm with mathematical averages and Dice similarity coefficients using the GelCompar II 6.5 software (Applied Maths NV). The Shannon-Wiener, Simpson, and Chao-1 alpha indices were calculated from the band patterns using the densitometry curves and then exported into a quantitative numeric matrix, relative to the band surface.

ANOVA test was performed using the Fisher LSD post hoc test at a significance level of *P* < 0.05. All the statistic analyses were performed in InfoStat programme [29].

### 2.5. Quantification of the Bacterial Community by Quantitative PCR (qPCR)

The abundances of 16S rRNA and *nifH* genes were quantified by real-time PCR (qPCR) using the primers 6S-27F/338R and POLF/POLR (Table S1), respectively [21, 22, 30]. The qPCR reaction was performed in a CFX96 Touch Real-Time PCR (Bio-Rad) equipment, and all measurements were performed using the SYBR Green approach. The PCR mixture was 12.5 *μ*l of the iQSYBR Green Supermix (Bio-Rad), 1 *μ*M of each primer, and 4 to 25 ng of DNA template, within a total volume of 25 *μ*l. The qPCR cycle for the 16S rRNA coding gene consisted of a denaturation step for 10 min at 95°C, 40 cycles for 15 s at 95°C, 30 s at 58°C, and 30 s at 72°C. The qPCR cycle for the *nifH* gene consisted of a denaturation step for 5 min at 95°C and 40 cycles for 10 s at 95°C, for 10 s at 59°C, and 30 s at 72°C. Product specificity was confirmed by melting curve analysis (58–95°C, 0.5°C per read, 5 s hold) and visualization in agarose gels, which showed specific product bands at the expected size of 180 bp for the 16S rRNA gene and 360 bp for the *nifH* gene.

For both genes, three replicates in duplicate were used. For the standard curve, triplicates were employed for every run using a known number of each gene from the genome of *Herbaspirillum seropedicae* SmR1 [31], from 6.62 × 10^1^ to 6.62 × 10^5^ copies of the *nifH* gene and from 1.99 × 10^3^ to 1.99 × 10^6^ copies of the 16S rRNA encoding gene.

For the standard curve, mass concentrations of standard DNA were converted into copy concentrations using the following equation [32]:

(1) $$\begin{array}{c} DNA\left(copy\right)=\frac{6.02\times 10^{23}\left(copy/mol\right)\times DNA\;amount\;\left(g\right)}{DNA\;length\left(bp\right)\times 660\left(g/mol/bp\right)}. \end{array}$$

For statistical analyses, an ANOVA test was performed using the InfoStat programme and in those circumstances where significant differences were confirmed, the means were compared using the Tukey test with a *P* < 0.05 [29].

### 2.6. Ion Torrent® High-Throughput Sequencing of the Bacterial 16S rRNA Coding Gene

#### 2.6.1. Sample Collection and DNA Extraction

For bacterial DNA extraction, same plant samples used in the DGGE experiments were employed. In this case, stems from four sweet sorghum plants were sampled and pooled from two different fields with contrasting N fertilization levels (0 and 100 kg N ha^−1^). Bacterial DNA extraction was performed following the protocol previously described [33], with the following modifications: stems were peeled with a sterilized scalpel, and 50 g of the inner stem tissues were homogenized in 300 ml of Milli-Q water. Bacterial DNA extraction from the enriched fraction was obtained using a CTAB bacterial DNA isolation method (Joint Genome Institute protocols/ http://1ofdmq2n8tc36m6i46scovo2e.wpengine.netdna-cdn.com/wp-content/uploads/2014/02/JGIBacterial-DNA-isolation-CTAB-Protocol-2012.pdf).

The purity of the extracted DNA was checked with the NanoDrop ND-1000 spectrophotometer (NanoDrop Technologies, Wilmington, DE, USA) (260/280 nm ratio), and it was quantified using an Agilent 2100 Bioanalyzer (Agilent Technologies, Santa Clara, CA, USA). The integrity of the DNA was also confirmed by electrophoresis in a 0.8% agarose gel with 1x TAE buffer.

#### 2.6.2. Sequencing and Data Analysis

PCR amplification of the V6 region of the 16S rRNA gene sequences was carried out using a pool of six forward and reverse degenerate primers each (Table S2) [34]. High-throughput sequencing of the amplicons was conducted using the Ion Torrent Personal Genome Machine (PGM) platform at the Genomic Department of the IIBCE (Uruguay). Raw sequencing reads were checked using the following quality criteria: (i) polyclonal reads with Ion Torrent Suite (5.03) were discarded, (ii) reads were trimmed to 90 bp and shorter ones were discarded, (iii) reads with an expected error rate equal to or greater than 1.0 using Usearch (Edgar, 2010) were also discarded, and (iv) reads that match with the *Sorghum bicolor* complete genome [35] using Bowtie 2 [36] were discarded. Downstream analysis followed the pipeline described by Pylro et al. [37]. A custom set of bash scripts was constructed to automatize the pipeline; these are available on the site https://github.com/mberacochea/sorghum-bicolor-M81E-16S. All sequences obtained in this study were deposited under the NCBI accession number PRJNA352426.

## 3. Results

### 3.1. Endophytic Bacterial Communities Associated with Different Tissues of Sweet Sorghum Plants

DNA was recovered from all the sweet sorghum samples (roots and stems) of cv. M81E grown in the field under different N fertilization levels (0 and 100 kg N ha^−1^). All DNA samples were used as templates for PCR amplification using primers based on general 16S rRNA coding genes, on specific 16S rRNA genes for Alpha-, Beta-, and Gammaproteobacteria, Actinobacteria, Firmicutes, and on the *nifH* gene (Table S1). DNA fragments of the expected size obtained using each set of primers were resolved by DGGE.

The results from the DGGE analysis of the total endophytic bacterial community based on the 16S rRNA gene amplicon are shown in Figure 1(a). The UPGMA-assisted cluster analysis of the DGGE gels revealed the endophytic communities clustered according to the organ analyzed (roots and stem) with 50.5% similarity (Figure 1(b)). Moreover, within the aforementioned organs, the communities are grouped with respect to the N fertilization level analyzed, with 61.3 and 66.4% similarities observed for the stems and roots, respectively. However, no distinct community structuring was observed between the lower and upper stems (Figure 1(b)).

When the whole endophytic community was analyzed by DGGE, alpha diversity indices (Simpson 1-D, Chao-1, and Shannon H) showed that the higher values were obtained for the root communities at the low N fertilization level, but no significant differences were observed among the treatments analyzed (Table S3).

The DNA bands were retrieved from the DGGE gels, reamplified, and sequenced (Figure 1(a)). BLASTn analyses revealed that all the identified genera belonged to the phylum Proteobacteria (Table S2), which was primarily represented by genera from the classes Betaproteobacteria (*Duganella*, *Aquabacterium*, *Bordetella*, and *Massilia*) and Gammaproteobacteria (*Pantoea*, *Salmonella*, *Klebsiella*, *Kosakonia*, *Pseudomonas*, *Serratia*, and *Stenotrophomonas*).

Interestingly, when the phyla Proteobacteria (classes Alpha, Beta, and Gamma), Firmicutes, and Actinobacteria were analyzed using specific primers based on the 16S rRNA gene amplicon (Table S1), the UPGMA-assisted cluster analysis of the DGGE gels revealed that the structure of those communities did not cluster according to the plant organ (roots and stem) or to the fertilization level analyzed (0 and 100 kg N ha^−1^) (data not shown). Selected bands retrieved from each DGGE gel are shown in Table S4.

Analysis of the Alphaproteobacteria DNA bands amplified using the specific primers and retrieved from the DGGE gels showed that only 45% of the sequences were related to the expected genera, such as *Agrobacterium*, *Ancylobacter*, *Brevundimonas*, and *Pleomorphomonas* (Table S4). In contrast, analysis of the Betaproteobacteria bands isolated from the DGGE gels, which were amplified using the specific primers, revealed that 100% of the sequences were related to the expected genera. From the Betaproteobacteria DGGE gel, only the genera *Massilia* and *Methyloversatilis* were identified (Table S4). Despite the specificity of the primers, none of the selected bands sequenced from the Gammaproteobacteria DGGE gels was assigned to a genus belonging to this class. By contrast, the identities of the bands retrieved from the Actinobacteria DGGE gels showed that 100% of the genera identified were related to the expected phyla, including *Curtobacterium*, *Microbacterium*, *Nocardia*, *and Sediminihabitans* (Table S4). Similarly, the sequence identities of the bands retrieved from the Firmicutes DGGE gel were also 100% related to the expected phyla, including *Bacillus*, *Macrococcus*, *Staphylococcus*, and *Exiguobacterium* (Table S4).

In contrast to the 16S rRNA-based data, analysis of the endophytic-diazotrophic bacterial community based on DGGE gels with the *nifH* gene amplicons showed that the stems produced a greater number of bands than the roots in both treatments analyzed (Figure 2(a)). In addition, the UPGMA-assisted cluster analysis of the endophytic-diazotrophic community structure showed that this community was clustered according to treatment (0 and 100 kg N ha^−1^) that exhibited 34.7% similarity, except for a single root replicate. Moreover, within these treatments, the communities were grouped according to the organs analyzed, with 51.2 and 37.8%, respectively. In particular, under low N fertilization conditions, the stem community separated into two groups (lower and upper stem samples) that exhibited 47.5% similarity (Figure 2(b)).

The diversity of diazotrophic bacterial communities was also evaluated based on the DGGE gels obtained. In this case, higher alpha index values were obtained in the stems from the high N fertilization condition. However, in all the treatments analyzed, no significant differences were observed (Table S5). Selected bands from the DGGE gels in which the diazotrophic community was analyzed were excised and reamplified, and the products were sequenced (Figure 2(a)). BLASTn analyses revealed that all the bands were closely related to *nifH* genes of Gammaproteobacteria (71%), Betaproteobacteria, and Cyanobacteria (4%), while 21% were related to unculturable bacteria (Table S5). From the last group, the first hit from the BLASTn analysis that matched to a culturable strain was also taken into account. In this case, all the sequences were closely associated with *nifH* genes from Gammaproteobacteria members (Table S5).

### 3.2. Quantification of the Endophytic and Diazotrophic-Endophytic Bacterial Communities

**Figure 3:**
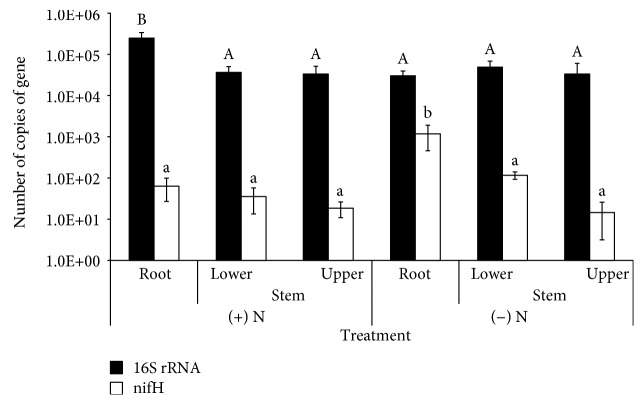
Quantification of 16S rRNA and *nifH* genes copies in samples taken from different organs of sweet sorghum plants (cv. M81E) grown in the field under high (+) and low (−) N fertilization (100 and 0 kg N ha^−1^, respectively). Means within two treatments that have the same letter are not significantly different by Tukey test with a *P* < 0.05.

The abundances of the bacterial endophytic and endophytic-diazotrophic communities were assessed by qPCR using the 16S rRNA and *nifH* genes. The standard curves for the 16S rRNA gene amplification showed a linear correlation (*R* ^2^) of 0.99, corresponding to a PCR efficiency of 90%; for the *nifH* gene, the linear correlation was 0.98, corresponding to a PCR efficiency of 110%. The 16S rRNA gene abundance varied from 3.0 × 10^4^ to 2.5 × 10^5^, while for the *nifH* gene, the number of copies varied from 1.9 × 10^1^ to 1.2 × 10^3^ (Figure 3).

The number of copies of the 16S rRNA gene in the roots of plants grown under high N fertilization (+N) conditions was significantly higher than in all other conditions analyzed (Figure 3). By contrast, the number of *nifH* gene copies varied markedly in the roots and stems (lower and upper) of plants grown under low N fertilization (−N) conditions, while no differences were observed within the plants grown under +N conditions (Figure 3). Indeed, under −N conditions, the abundance of the *nifH* genes was significantly higher in the roots than in the other organs analyzed.

### 3.3. Bacterial Community Composition

After quality control, the number of retained reads was 41,736 and 44,890 for the DNA samples from plants grown under +N and −N conditions, respectively. Rarefaction curves were used to assess OTU richness and showed that an asymptote was reached for both treatments analyzed, with a higher number of OTUs observed in the +N treatment (Figure S1).

**Figure 4:**
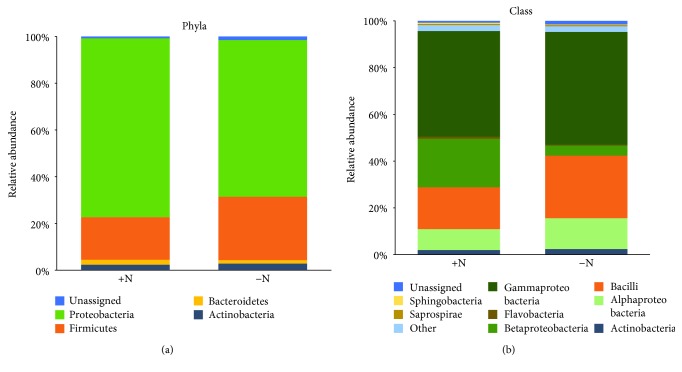
Taxonomic composition of the 16S rRNA samples associated with sweet sorghum plants (cv. M81E) grown in the field under different N fertilization levels (+/−N). Relative abundance (over 0.5%) of the bacteria at the level of (a) phylum and (b) class.

The relative abundance of the microbial clades at two taxonomic levels (phylum and class) is summarized in Figure 4. At the phylum level, OTUs related to Proteobacteria and Firmicutes dominated both treatments analyzed, with relative abundances of 75/65 and 18/27% in each treatment (+/−N), respectively. In addition, Actinobacteria and Bacteroidetes OTUs accounted for approximately 2% of the relative bacterial abundances in both treatments.

An analysis at the class level showed that in the +N treatment, Gammaproteobacteria was the most abundant class (45%), followed by Betaproteobacteria (21%), Bacilli (18%), Alphaproteobacteria (8.9%), and Actinobacteria (2%). The most abundant class in the −N treatment was also Gammaproteobacteria (48%), followed by Bacilli (27%), Alphaproteobacteria (13%), Betaproteobacteria (4.2%), and Actinobacteria (2.4%) (Figure 4).

Analysis of OTUs exhibiting a 10-fold change in abundance between treatments showed that OTUs that were most affected by the N fertilization treatment were members of the Beta- and Gammaproteobacteria, as well as Firmicutes. Within the Betaproteobacteria class, the OTUs associated with the genus *Herbaspirillum* increased from 0.1 to 5.0% after the N fertilization treatment. For the Gammaproteobacteria, large changes were observed in OTUs related to the genus *Erwinia*, showing a decrease from 21.6 to 1.1%, while *Pseudomonas* increased from 1.3 to 17.9% after the N fertilization treatment. Finally, within Firmicutes, OTUs related to the genus *Bacillus* decreased from 14.8 to 8.6% in response to fertilization with 100 kg N ha^−1^.

## 4. Discussion

Understanding the effects of agronomical practices, e.g., chemical fertilization, on plant microbiota is necessary to optimize plant-microbiota communities with the aim of improving agronomical sustainability.

In this work, different culture-independent methods were used to evaluate the impact of chemical N fertilization on the endophytic and diazotrophic-endophytic communities associated with the commercial sweet sorghum cv. M81E grown under field conditions. These methodologies are powerful tools that have greatly contributed to identifying the microbial composition and diversity in a wide range of ecosystems, including the interior of plant tissues [38–41].

Our results demonstrated that N fertilization in the field influenced the structure but not the diversity of the endophytic bacterial community within each organ analyzed (roots and stems). This result agrees with a previous study, in which the structures of sorghum stem and root communities were shown to be significantly different [42]. Moreover, the effects of chemical fertilization on the endophytic bacterial community structure were reported for several grasses, such as *Dactylis glomerata*, *Festuca rubra*, and *Lolium perenne* [43, 44]. Different selective processes act together during the recruitment of bacteria that finally colonize the internal tissues of plants [8]. Thus, the combination of N fertilization and the specific plant organ features (e.g., high sugar content) could be the primary factors that influenced the bacterial endophyte structure observed in this study.

Additionally, we observed that the bacterial abundance in the stems of both treatments analyzed was statistically the same and was not influenced by the N fertilization treatment. Nevertheless, the bacterial abundance on roots was increased in response to N fertilization, a case where the diversity was lower but not significantly so. Because root tissues are the primary entry point for bacteria, N fertilization may directly affect the physiological state of roots and the bacteria in the vicinity that can effectively infect the root internal tissues as a consequence [39, 45].

With respect to the bacteria identified, the results showed that the 16S rRNA gene sequences from bands retrieved from DGGE were all related to Proteobacteria, as was previously reported for arable sweet sorghum [46]. Moreover, high-throughput sequencing analysis based on the 16S rRNA gene from the potential endophytic bacterial community also showed that most of the OTUs obtained from both treatments were also related to Proteobacteria. These results agree with those obtained by Maropola et al. [42], who observed that Proteobacteria, in addition to Firmicutes and Actinobacteria, were the most dominant phyla in both communities analyzed (roots and stems) of sweet sorghum plants.

Interestingly, when Proteobacteria (Alpha and Beta), Actinobacteria, and Firmicutes phyla were analyzed by DGGE, several genera were identified, in agreement with previous a study in which the culturable endophytic community associated with sweet sorghum was analyzed [19, 47, 48]. Moreover, from the DGGE analysis, we showed that the genera *Massilia* from the class Betaproteobacteria, as well as *Bacillus* from the phylum Firmicutes, were well represented. Species of both genera are common inhabitants of the inner tissues of various species of plants, where they play an important role in plant protection and growth promotion [4, 8, 14].

With respect to the effect of the chemical fertilization on the composition of the bacterial community, it was interesting to observe that the Betaproteobacteria, as well as members of the class Bacilli, had large shifts in the number of OTUs present after the application of the N fertilizer. OTUs that increased in abundance in the presence of N fertilizer were associated with the genera *Pseudomonas* and *Herbaspirillum*, while the OTUs that decreased were associated with the genera *Bacillus* and *Erwinia*. The association of representatives of these genera with different plants (including sweet sorghum) has previously been described [14]. Our results support the hypothesis that the physiological state of the plant modulates the bacterial microbiota composition recruiting specific bacteria, a phenomenon that may play a key role in promoting plant health and growth [8, 39, 45]. Further experiments are needed to determine the specific functions of the identified bacterial genera in the microbiota of sweet sorghum.

In addition, we observed that the level of N fertilization was the primary factor affecting the structure of the diazotrophic-endophytic bacterial community, but it did not significantly affect its diversity. In addition, the abundances of the *nifH* gene in the internal tissues of both analyzed treatments were not significantly different, except for the roots of plants grown without N fertilization. In the latter case, the abundance of the diazotrophic-endophytic bacterial community was higher and less diverse than that in the roots of plants grown without N fertilization. These results also support the hypothesis that under certain conditions, a specific endophytic community is recruited from a pool of opportunistic bacteria present in the soil and that those most competitive can infect and survive within the plant tissue environment [8, 42, 46]. In this study, the absence of N fertilization may have prompted plants to recruit diazotrophic bacteria, which may contribute to the plant growth promotion via the BNF process. This is supported by the observation that positive BNF was detected in the roots of sweet sorghum plants by an acetylene reduction assay (ARA) [48]. Moreover, our results are consistent with previous studies in which the *nifH* gene abundance of sweet sorghum, maize, and rice treated with different levels of nitrogen fertilizer was studied [49–51]. However, it should be noted that because the present study was based on the extraction of total DNA from the roots and stems of sorghum plants, we cannot assume that the *nifH* genes were actually active.

The identities of the *nifH* amplicon sequences retrieved from the DGGE gels belonged to the phyla Cyanobacteria and Proteobacteria. Within the Proteobacteria, as was observed in the 16S rRNA gene analysis, the most abundant class detected was the Gammaproteobacteria. Similar results were obtained when the diversity of the *nifH* gene pools of sweet sorghum was studied, but in this case, the most abundant classes affected by the chemical N fertilization were Alpha- and Betaproteobacteria [49]. Is interesting to note that within the Gammaproteobacteria, most of the sequences were from the genera *Enterobacter* and *Klebsiella*, which are well described as diazotrophic-endophytic plant growth promoters [52, 53] and as being associated with sweet sorghum plants [19, 48]. These results stress the role that these genera may play as PGP diazotrophs in the microbiota associated with sweet sorghum plants.

## 5. Conclusions

The results obtained in our study showed that the application of N fertilizer affected the structure, abundance, and composition of the endophytic bacterial communities associated with sweet sorghum plants. This effect was pronounced in the roots of both bacterial communities analyzed and may have depended of the physiological state of the plants. Moreover, specific bacterial classes and genera increased or decreased when the fertilizer was applied. The data obtained in this study contribute to a better understanding of the effects of different agronomical practices on the microbiota associated with this important crop, which may help improve its sustainability.

## Data Availability

The data used to support the findings of this study are available from the corresponding author upon request.

## Abbreviations

DGGE: Denaturing gradient gel electrophoresis
PGPB: Plant growth-promoting bacteria
BNF: Biological nitrogen fixation.

## Supplementary Materials

Supplementary Materials Table S1: primers used for PCR amplification. Table S2: primers used for Ion Torrent pyrosequencing analysis. Table S3: alpha diversity indices. Statistical analysis of the total endophytic and diazotrophic-endophytic bacterial community associated with sweet sorghum cv. M81E, grown under different N fertilization levels (+/−N). Table S4: identification by NCBI BLASTn of 16S rRNA sequences retrieved from DGGE bands. Table S5: identification by NCBI BLASTx of nifH sequences retrieved from DGGE bands. Figure S1: rarefactions curves of sweet sorghum samples treated with high and low N fertilization levels (+N and –N, respectively).
