# Supplementary material for: The Endophytic Bacterial Microbiota Associated with Sweet Sorghum (Sorghum bicolor) Is Modulated by the Application of Chemical N Fertilizer to the Field
Source: Int J Genomics. 2018 Sep 30;2018:7403670. doi: 10.1155/2018/7403670 (PMC6186372; doi:10.1155/2018/7403670)

Table S1. Primers used for PCR amplification

Name	Primer Sequence (5'-3')	Gene target	Taxon target	Reference
**First PCR round DGGE analysis**				
FGPH19	TACGGCAARGGTGGNATHG	*nifH*	Diazotrophic	(Simonet et al. 1991)
POLR	ATSGCCATCATYTCRCCGGA	*nifH*	Diazotrophic	(Poly et al. 2001)
799F	AACMGGATTAGATACCCKG	16S rRNA	Bacteria	(Chelius and Triplett 2001)
1492R	TACGGYTACCTTGTTACGACTT	16S rRNA	Bacteria	(Chelius and Triplett 2001)
F203α	CCGCATACGCCCTACGGGGGAAAGATTTAT	16S rRNA	Alphaproteobacteria	(Gomes et al. 2001)
F948β	CGCACAAGCGGTGGATGA	16S rRNA	Betaproteobacteria	(Gomes et al. 2001)
F243HCG	GGATGAGCCCGCGGCCTA	16S rRNA	Actinobacteria	(Heuer et al. 1997)
BACF	GGGAAACCGGGGCTAATACCGGAT	16S rRNA	Firmicutes	(Garbeva et al. 2003)
**Second PCR round DGGE analysis**				
POLF-GC	CGCCCGCCGCGCCCCGCGCCCGGCCCGCCCCCG CCCCTGCGAYCCSAARGCBGACTC	*nifH*	Diazotrophic	(Poly et al. 2001)
AQER	GACGATGTAGATITCCTG	*nifH*	Diazotrophic	(Poly et al. 2001)
F968-GC	CGCCCGGGGCGCGCCCCGGGCGGGGCGGGGGC ACGGGGGGAACGAAGAACCTTAC	*16S rRNA*	Bacteria	(Heuer et al. 1999)
R1401	CGGTGTGTACAAGACCC	*16S rRNA*	Bacteria	(Heuer et al. 1997)
**qPCR analysis**				
POLR	ATSGCCATCATYTCRCCGGA	*nifH*	Diazotrophic	(Poly et al. 2001)
POLF	TGCGAYCCSAARGCBGACTC	*nifH*	Diazotrophic	(Poly et al. 2001)
6S-27F	AGAGTTTGATCCTGGCTCAG	16S rRNA	Bacteria	Bulgari et al., 2014
338R	GCTGCCTCCCGTAGGAGT	16S rRNA	Bacteria	Bulgari et al., 2014

Table 2. Primers used for Ion Torrent pyrosequencing analysis.

Primer	Primer sequence (5´-3´)	Reference
967F-PP	CNACGCGAAGAACCTTANC	(Jünemann et al. 2012)
967F-UC1	CAACGCGAAAAACCTTACC	(Jünemann et al. 2012)
967F-UC2	CAACGCGCAGAACCTTACC	(Jünemann et al. 2012)
967F-UC3	ATACGCGARGAACCTTACC	(Jünemann et al. 2012)
967F-AQ	CTAACCGANGAACCTYACC	(Jünemann et al. 2012)
1046R	CGACAGCCATGCANCACCT	(Jünemann et al. 2012)
1046R-PP	CGACAACCATGCANCACCT	(Jünemann et al. 2012)
1046R-AQ1	CGACGGCCATGCANCACCT	(Jünemann et al. 2012)
1046R-AQ2	CGACGACCATGCANCACCT	(Jünemann et al. 2012)

Table S3. Alpha diversity indices. Statistical analysis of the total endophytic and diazotrophic endophytic bacterial community associated with sweet sorghum cv. M81E, grown under different N-fertilization levels (+/-N).

Treatment/ N- fertilization level[*]	Endophytic bacteria			Diazotrophic endophytic bacteria		
	Chao-1 (richness)	Simpson 1-D	Shannon H	Chao-1 (richness)	Simpson 1-D	Shannon H
**R –N**	21.55	0.91a	2.69a	5.67	0.41a	0.96a
**R +N**	16.41	0.87a	2.33a	8.33	0.71a	1.51a
**LS –N**	17.16	0.84a	2.25a	7.66	0.78a	1.71a
**LS +N**	20.33	0.88a	2.54a	9.66	0.82a	1.95a
**US –N**	20.66	0.90a	2.63a	9.33	0.73a	1.66a
**US +N**	20.33	0.89a	2.55a	9.66	0.81a	1.91a

[*]R: root, LS: low stem, US: upper stem, +/-N: high and low N-fertilization level. Means within two treatments that have the same letter are not significantly different by Fisher LSD test with a $P<0.05$

Table S4. Identification by NCBI BlastN of 16S rRNA sequences retrieved from DGGE bands.

	Bands	Accession number NCBI	Closest match	% Identity
		**From the general *16S RNAr* DGGE gel**		
**Betaproteobacteria**	**B7**	NR_025770.1	*Duganella violaceinigra* YIM 31327	99%
	**B14**	NR_108739.1	*Aquabacterium limnoticum*ABP-4	97%
	**B20**	NR_027537.1	*Bordetella hinzii* R-39474	97%
	**B21**	NR_137346.1	*Massilia arvi* THG-RS2O	96%
**Gammaproteobacteria**	**B8, B10**	NR_103936.1	*Klebsiella pneumoniae* DMS 30104	99%
	**B23**	NR_112008.1	*Serratia liquefaciens* JCM1245	100%
	**B9, B24, B33**	NR_043883.1	*Pantoea dispersa*LMG 2603	99%
	**B1, B2, B3, B29, B30**	NR_041973.1	*Pantoea cypripedii* DSM 3873	99%
	**B4, B5, B31**	NR_074910.1	*Salmonella enterica* serovar Typhimurium LT21	99%
	**B25**	NR_116403.1	*Kosakonia radicincitans* Ah-143	99%
	**B26**	NR_137380.1	*Pantoea intestinalis strain* 29Y89B	90%
	**B27, B38**	NR_116748.1	*Pantoea brenneri* LMG 5343	99%
	**B28**	NR_114481.1	*Pseudomonas tolaasii* ATCC 33618	94%
	**B32**	NR_114507.1	*Klebsiella pneumoniae subsp. rhinoscleromatis ATCC 13884*	99%
	**B34, B37**	NR_026045.1	*Pantoea ananatis* LMG 1846	99%
	**B36**	NR_113648.1	*Stenotrophomonas maltophilia* NBRC 14161	98%
		**From the Alphaproteobacteria DGGE gel**		
	**B12a**	NR_026519.1	*Agrobacterium larrymoorei* AF3.10	99%
	**B13a**	NR_044393.1	*Ancylobacter oerskovii* NS05	94%
	**B17a**	NR_037106.1	*Brevundimonas variabilis* CB17	99%
	**B18a, B19a**	NR_114056.1	*Pleomorphomonas oryzae* NBRC 102288	98%
		**From the Betaproteobacteria DGGE gel**		
	**B1b, B2b, B4b**	NR_043308.1	*Massilia albidiflava* 45	98%
	**B5b, B8b, B9b, B11b**	NR_137346.1	*Massilia arvi* THG-RS2O	99%

**B7b**	NR_136517.1	*Methyloversatilis discipulorum* FAM1	97%
**From the Actinobacteria DGGE gel**			
**B7ac**	NR_114322.1	*Sediminihabitans luteus*H97-3	96%
**B9ac**	NR_104839.1	*Curtobacterium oceanosedimentum* ATCC31317	99%
**B13ac**	NR_135869.1	*Microbacterium proteolyticum*	99%
**B15ac**	NR_116334.1	*Nocardia mikamii* W8061	100%
**From the Firmicutes DGGE gel**			
**B1f**	NR_118445.1	*Bacillus circulans ATCC 4513*	97%
**B2f**	NR_117050.1	*Bacillus kochii WCC 4582*	98%
**B3f, B10f**	NR_108906.1	*Bacillus eiseniae A1-2*	98%
**B4f**	NR_036847.1	*Macrococcus brunensis* CCM 4811	100%
**B5f**	NR_118534.1	*Exiguobacterium himgiriensis* K22-26	99%
**B6f**	NR_036904.1	*Staphylococcus epidermidis* Fussel	99%
**B8f**	NR_113585.1	*Exiguobacterium acetylicum NBRC 12146*	99%
**B11f**	NR_036828.1	*Staphylococcus capitis* JCM 2420	100%

Table S5. Identification by NCBI Blastx of *nifH* sequences retrieved from DGGE bands.

**Bands**	**Accession number NCBI**	**Closest match**	**% identity**	**Accession number NCBI**	**Closest match (culturable)**	**% identity**	**Phylogenetic position (Class[a]/Order)**
**B1n**	GU056207.1	*Oscillatoria* sp. MMG-2	96%				Cyanobacteria, Occilatoriales
**B4n**	FJ593869.1	*Pantoea* sp. EC080527_02	97%				γ/Enterobacteriales
**B5n**	KC989924.1	*Enterobacter* sp. R4-368	98%				γ/Enterobacteriales
**B6n, B23n, B30n**	HQ204226.1	*Enterobacter* sacchari HX148S	98%-99%				γ/Enterobacteriales
**B7n**	KF872961.1	Uncultured bacterium clone IFRpool-70	99%	HQ204232.1	*Enterobacter* sp. PG132S	94%	γ/Enterobacteriales
**B8n**	KF872888.1	Uncultured bacterium clone ABFRpool-29	100%	HQ204226.1	*Enterobacter sacchari* HX148S	99%	γ/Enterobacteriales
**B9n**	LC049050.1	*Klebsiella variicola*	100%				γ/Enterobacteriales
**B10n**	KF732646.1	*Klebsiella variicola* DX120E	99%				γ/Enterobacteriales
**B11n, B15n**	HQ404304.1	*Klebsiella pneumoniae* NG14	99%				γ/Enterobacteriales
**B12n**	FJ593869.1	*Pantoea* sp. EC080527_02	99%				γ/Enterobacteriales
**B13n, B17n, B21n**	HQ204258.1	*Enterobacter sacchari* NN143E	99%				γ/Enterobacteriales
**B14n**	HQ204222.1	*Klebsiella* sp. CZ150S	99%				γ/Enterobacteriales
**B16n**	HQ204258.1	*Enterobacter sacchari* NN143E	76%				γ/Enterobacteriales
**B18n**	EF438102.1	Uncultured marine microorganism clone C11-16	88%	CP001614.2	*Teredinibacter turnerae* T7901	80%	γ/Alteromonadales
**B19n**	AY231580.1	*Ideonella* sp. Long 7	98%				β/Burkholderiales
**B20n**	AF331982.1	Uncultured bacterium clone g1(102)	85%	CP010415.1	*Azotobacter chroococcum* NCIMB 8003	84%	γ/ Pseudomonadales
**B22n**	KC989924.1	*Enterobacter* sp. R4-368	99%				γ/ Enterobacteriales

**B24n**	KF872888.1	Uncultured bacterium clone ABFRpool-29	96%	HQ204258.1	*Enterobacter sacchari* NN143E	95%	γ/ Enterobacteriales
**B25n**	HQ204233.1	*Enterobacter* sp. QZ25S	99%				γ/ Enterobacteriales
**B27n**	CP011636.1	*Klebsiella oxytoca* CAV1374	99%				γ/ Enterobacteriales
**B31n**	CP007215.2	*Enterobacter sacchari* SP1	97%				γ/ Enterobacteriales
**B32n**	KM885223.1	Uncultured microorganism clone L-49	99%	FJ593868.1	*Enterobacter* sp. MTP_05051217	99%	γ/ Enterobacteriales

[a]β: Betaprotebacteria; γ: Gammaprotebacteria.

Figure S1. Rarefactions curves of sweet sorghum samples treated with high and low N-fertilization levels (+N and –N respectively).

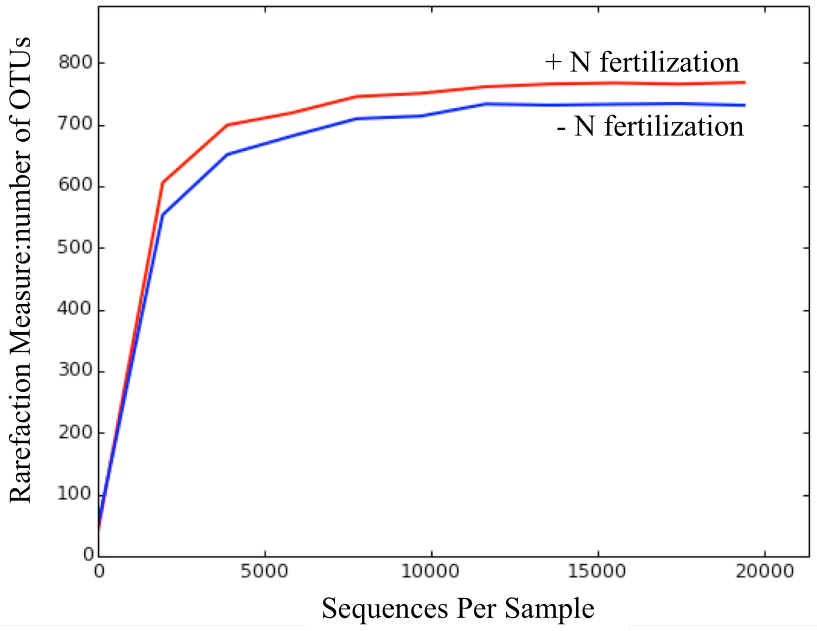

Supplement: Supplementary Materials — Table S1: primers used for PCR amplification. Table S2: primers used for Ion Torrent pyrosequencing analysis. Table S3: alpha diversity indices. Statistical analysis of the total endophytic and diazotrophic-endophytic bacterial community associated with sweet sorghum cv. M81E, grown under different N fertilization levels (+/−N). Table S4: identification by NCBI BLASTn of 16S rRNA sequences retrieved from DGGE bands. Table S5: identification by NCBI BLASTx of nifH sequences retrieved from DGGE bands. Figure S1: rarefactions curves of sweet sorghum samples treated with high and low N fertilization levels (+N and –N, respectively). [file 7403670.f1.pdf]
